# Psychometric properties of the Persian version of the quality of recovery-15 questionnaire

**DOI:** 10.1186/s41687-021-00351-9

**Published:** 2021-10-23

**Authors:** Hooman Shahsavari, Golnar Ghane, Shahrzad Ghiyasvandian, Masoumeh Zakerimoghadam, Fatemeh Najafi

**Affiliations:** Department of Medical Surgical Nursing, School of Nursing and Midwifery, Tehran University of Medical Sciences, Nosrat St., Tohid Sq., Tehran, Iran

**Keywords:** Surgery, Quality of recovery, Validity, Reliability, Psychometric properties

## Abstract

**Background:**

Recovery after surgery is a complex process since it depends on many factors, such as the patient’s sex, age, surgery type, and presence of other diseases. This study aimed to translate and evaluate the psychometric properties of the Persian version of the quality of recovery-15 (QoR-15) questionnaire in Iranian patients undergoing surgery.

**Methods:**

The Persian version of the QoR-15 questionnaire was developed after translating and culturally validating the instrument. Content validity was assessed with a sample of clinicians (n = 15) and face validity was assessed in a sample of patients (n = 15) undergoing elective surgery. The final questionnaire was completed by 450 patients (n = 450) 24 h after surgery. Construct validity was assessed using exploratory factor analysis in patients (N = 250). Convergence and divergent validity were also assessed. Internal consistency was assessed using Cronbach's alpha and construct reliability was also assessed. Test–retest reliability was assessed on a randomly selected sub sample of 50 patients. Finally, the questionnaire was completed by a further sample of 200 patients 24 h after surgery and construct validity was assessed using confirmatory factor analysis.

**Results:**

According to Lawshe, all items received at least an acceptable ratio for content validity ratio (CVR). Item content validity index (I-CVI) of each item was greater than 0.79. Construct validity indicated good fit statistics in the five components of CFA, and CFI was > 0.93. The reliability of the QoR-15 questionnaire was acceptable based on Cronbach’s alpha score (> 0.001), test–retest reliability value (0.81), and CR (> 0.7).

**Conclusion:**

The Persian version of the QoR-15 questionnaire was equivalent to the original one regarding both conceptual and linguistic aspects. This study also confirmed the validity and reliability of the Persian version of the QoR-15 questionnaire. Therefore, the Persian version of the QoR-15 questionnaire can be a suitable and brief instrument to assess the recovery quality in Iranian patients undergoing surgery.

## Background

Today, surgery is considered the main treatment method in many diseases, so that the number of patients in need of complex surgeries for treatment is increasing. One of the things that should be considered after surgery is to evaluate the patient's recovery after surgery and the duration of hospitalization. Recently, more efforts have been made to accelerate the patient's return to normal preoperative activities and reduce the cost of hospitalization [1, 2].

Postoperative recovery and anesthesia is a complex process that depends on the patient's characteristics, surgery, anesthesia, as well as the presence of any preoperative disorders and adverse complications after surgery [3]. Traditionally, postoperative patient recovery assessment focuses on measurements of physiological factors, morbidity, mortality, postoperative adverse events, physiological changes in patients, and readmission rate. Although these parameters are important and should be measured, these data present only one aspect of the patient's recovery. The patient's ability and feeling to continue normal activities after surgery and anesthesia is also an important indicator in a successful recovery experience [4].

The quality of recovery(QoR), in addition to physiological and physical recovery, depends on the patients themselves, their mental perceptions, including emotional state and perceived physiological support [1, 2]. So that the basis of recovery after surgery is to regain mental and physical health. While in clinical evaluations, an examination from the patient's perspective is ignored [2, 3]. Therefore, measuring the quality of postoperative recovery from the patient's point of view requires evaluating multiple patient-centered outcomes.

To assess QoR from the patients' perspective, different comprehensive, appropriate, and relevant QoR questionnaires have been developed [5]. One of them, the QoR-40 questionnaire, is a generic and complete postoperative recovery instrument developed by Myles et al., in 1999 [6]. The QoR-40 questionnaire has 40 items categorized in five dimensions, including patient's support, comfort, emotions, physical independence, and pain. It has been translated and validated in different languages. It also has been validated and psychometrically assessed in Iran [7]. Although the instrument is well-validated, its feasibility in some clinical situations is problematic and controversial. Regarding its ease of use, studies reported patients' poor cooperation in completing the questionnaire after surgery[8]. Therefore, the QoR-15 questionnaire has been developed to assess recovery in patients by resorting to a more simplified and patient-friendly technique. Despite the brevity of the QoR-15 questionnaire, the quality of the instrument is not hurt [5, 9].

The QoR-15 questionnaire is developed by Stark et al., in 2013 [10]. The QoR-15 questionnaire is based on the original version and covers well all dimensions of postoperative recovery. It estimates QoR in five dimensions of pain, physical well-being, physical independence, psychological support, and emotional state. The instrument is based on 15 questions, and its total score ranges from 0 to 150 where high scores indicate good QoR. The original version of the QoR-15 questionnaire is a valid one with good validity, reliability, responsiveness, and feasibility [10], allowing its wider clinical application [5].

The QoR-15 questionnaire was first translated and validated in Danish [11]. Recently, the instrument has been translated and validated in Portuguese, Swedish, and Chinese [12–14]. The careful evaluation of patients depends on a careful assessment and deeper understanding of the context, culture, language, and ethnicity of individuals. Therefore, psychometrics is essential to reduce misunderstanding and have reliable instruments among multinational and multicultural populations. The instrument has been modified using many languages and cultural adaptations, leading to a higher possibility for investigating and comparing different populations at the international level. However, there is no validated Persian translation for the QoR-15 questionnaire.

## Methods

### Aim and Study design

The present study aimed at translating the QoR-15 questionnaire into Persian as well as exploring psychometric properties of the Persian version of this instrument in Iranian patients undergoing surgery. The quantitative approach was implemented in this psychometric research and methodological study.

### Study setting

We conducted this study in hospitals of Tehran University of Medical Science(TUMS) from May to December 2020. The study protocol was approved by a research ethics committee of the Tehran University of Medical Science(TUMS) (Number: IR.TUMS.FNM.REC.1398.217).

### Patients and data collection

Patients were selected from those scheduled for general and orthopedics surgery under general anesthesia. The inclusion criteria were as follows: being over the age of 18 years old, undergoing surgery within the last 24 or 48 h, being admitted to the surgical and orthopedic units of the hospitals of TUMS, being alert and able to communicate, being able to speak in the Persian language, and having the willingness to participate in the study. Patients with cognitive disorders, those who aged younger than 18 years or older than 80 years, and those with alcoholism and drug abuse were excluded from the study.

### Sampling method

We devised a purposive sampling method, to recruit patients undergoing surgery, meeting the eligibility criteria from surgical and orthopedic wards in hospitals of Tehran University of Medical Sciences. The study aims were explained to eligible patients. Then, the written informed consent was taken from all the patients. Patients completed the questionnaire 24 h after the surgery.

According to Munro (2005), the required number of respondents for exploratory factor analysis (EFA) should be between 3 and 10 participants per item or a total of 100 to 200 respondents [15]. In the present study, 450 patients were included. To evaluate construct validity, EFA were performed on the data collected from 250 patients. Confirmatory factor analysis(CFA) was also assessed in 200 patients.

### Measure

The QoR-15 questionnaire was completed 24 h after surgery by the patients. Patients’ demographic information, such as age, gender, time of surgery, and duration of hospitalization were retrieved from the medical records of the patients.

#### The QoR-15 questionnaire

The QoR-15 questionnaire is a 15-question survey about the patients’ health status. It measures patients’ satisfaction and QoR in the following dimensions, namely well-being, nausea, pain, and sleep. Rating of the items is done using an 11-point Likert scale ranging from 0–10. The QoR-15 questionnaire has a maximum score of 150, indicating excellent recovery.

This questionnaire has two parts. The first part consists of 10 items on patient’s different emotions and abilities (i.e. the ability to breathe easily, ability to enjoy food, feeling alert and vitality, having a good and enough sleep, ability to do personal activities, ability to interact with the family and friends, being supported the by nurses and doctors, ability to return to normal life activities, feeling comfortable and in control, and feeling of satisfaction and happiness), and the second part includes 5 items on the degree of having symptoms (moderate pain, severe pain, nausea and vomiting, feeling of concern and anxiety, and feeling of discomfort and depression) [10]. The questionnaire was provided to the patients 24 h after the surgery. If the patient was discharged from the hospital in 24 h, the QoR-15 questionnaire was completed by telephone call.

### Data analysis

We evaluated the Persian version of the QoR-15 questionnaire by determining its face validity, content validity, convergent validity, divergent validity, construct validity, and reliability. Validity describes the accuracy of the questionnaire. In order to assess the constructive validity, EFA and CFA, convergence validity, divergent validity, and known-groups comparison were used [18].

SPSS-AMOS (version 24) was used for data analysis. Univariate and multivariate data distributions were examined separately to study the normal distribution and scatter data. The existence of multivariate scatter data and multivariate normal distribution were investigated using Mahalanobis d-squared method (P < 0.001) and Mardia’s coefficient (> 20).

### Translation procedure

In this study, the quality of recovery-15 questionnaire was translated and tested from English to Persian using Sousa et al. (2011) seven-step translation and testing process (Table 1). In this way, a four-stage translation process returns with a two-stage test for its psychometric properties were used (the sixth stage of the translation process and test usually do not) [16]. Before starting the translation process, we obtained permission from the original author of the QoR-15 questionnaire to translate and test it psychometrically in Iranian patients through email.

**Table 1:** Steps of translation, adaptation and validation of instruments

Steps	Definition
Step 1	Translation of the original instrument into the target language (forward translation or one-way translation)
Step 2	comparison of the two translated versions of the instrument (TL1 and TL2): synthesis
Step 3	blind back-translation (blind backward translation or blind double translation) of the preliminary initial translated version of the instrument
Step 4	comparison of the two back-translated versions of the instrument (B-TL1 and B-TL2): synthesis II
Step 5	pilot testing of the pre-final version of the instrument in the target language with a monolingual sample: cognitive debriefing
Step 6	preliminary psychometric testing of the pre-final version of the translated instrument with a bilingual sample. This step is rarely used
Step 7	full psychometric testing of the pre-final version of the translated instrument in a sample of the target population

In the first phase of the translation process, two bilingual and bicultural translators translated the instrument from English into Persian [16]. One of the translators had familiarity with medical terms and also had enough knowledge about the terms of surgery, quality of recovery, caring, and all content of this instrument. Then, in the second phase, the two proposed translations were matched and merged to have a unified Persian instrument. In the third phase, two English native translators back-translated the instrument into English blindly [16]. Translators were not allowed to see the original version of the QoR-15 questionnaire. In the fourth phase, the multidisciplinary committee (methodologist, nursing staff, surgeon, anesthesiologist, clinical psychologists, and translators) compared the items and the format of the returned translations with those of the original one [16]. We removed any ambiguities, differences, grammatical errors, and other detected problems. Before finalizing the instrument, we emailed the translated version to the original author for validation.

In the fifth phase, the pre-final version was assessed by an expert group (content validity). Also, the face validity of the QoR-15 questionnaire was assessed from the patients’ perspective. They evaluated the items using a clear or unclear dual scale and provided suggestions to clarify the language of items. More details of this process in the face and content validity section were presented. A final Persian version of the QoR-15 questionnaire was obtained after observing all of the criteria. This version of the QoR-15 was distributed among 450 patients to examine its reliability and validity. The final questionnaire was completed by 250 patients (n = 250) 24 h after surgery and construct validity was assessed using EFA. Convergence and divergent validity and, internal consistency with using Cronbach's alpha were also assessed. Finally, the questionnaire was completed by a further sample of 200 patients 24 h after surgery and construct validity was assessed using CFA.

### Content validity

To test the content validity [17], we used a group of healthcare professionals who were knowledgeable about the care of patients after surgery in recovery and surgical units. Qualitative assessment in this step was done by recruiting 15 experts (nine nursing staff, two anesthesiologists, two clinical psychologists, one methodologist, and one surgeon). Experts assessed and commented on the item wording, item allocation, and scaling of the items. Then, we revised the QoR-15 questionnaire base on their comments and suggestions.

Quantitative assessment was done by calculating the content validity ratio (CVR) and content validity index (CVI) of the items. CVR indicates whether the item is essential or not based on the perspective of the professional experts. For this purpose, 15 experts were asked to rate the essentiality of the QoR-15 items based on a 3-point Likert scale (i.e. not essential: 1, useful but not essential: 2, and essential: 3). CVR of each item was calculated by using the following formula:

$${\text{CVR = }}\left[ {{\text{ne }} - \left( {{\text{N/2}}} \right)} \right]/\left( {{\text{N/2}}} \right) .$$

In the above formula, N and ne are equal to the total number of experts and the number of experts who score the intended item as essential, respectively [17].

CVI is the most commonly used method to calculate content validity quantitatively. There are two kinds of CVI, namely Item-CVI (I-CVI) and Scale-level CVI (S-CVI). CVI also shows the degree to which the items of the intended scale are simple, relevant, and clear. We asked the same 15 panelists to rate the relevance of items of the QoR-15 questionnaire by using a 4-point Likert scale (i.e. 1 = not relevant, 2 = somewhat relevant, 3 = quite relevant, and 4 = highly relevant). I-CVI of each item is calculated by dividing the number of panelists who had rated that item as 3 or 4 by the total number of the panelists. The items which acquire an ICVI value of 0.79 or greater rate are appropriate [18, 19].

### Face validity

The face validity of the QoR-15 questionnaire was assessed from the patients’ perspective using both qualitative and quantitative approaches [20, 21].

To assess the QoR-15 questionnaire qualitatively, 15 patients undergoing elective surgery via general anesthesia were asked to assess and comment on the appropriateness, difficulty, relevance, and ambiguity of the items. The time needed for completing the scale was determined in this step. Based on patients’ feedbacks, the scale was revised to enhance its clarity and reduce its ambiguity [21].

Quantitative assessment was done by applying the item impact technique. The same 15 patients in the qualitative phase were asked to rate the importance of the items using a five-point Likert scale (1 = not important to 5 = completely important). The impact score of each item was determined by calculating the importance frequency:

$${\text{Impact Score}} = {\text{Frequency (\%)}}\times {\text{Importance}} .$$

In this formula, frequency is equal to the number of patients who ascribed a score of four or five to the intended item, and importance is equal to scores four or five. If the impact score of an item was greater than 1.5, the item is considered suitable [21].

### Construct validity

The psychometric analysis of the questionnaire was done as follows:

The construct validity was assessed using exploratory factor analysis (EFA; N = 250) and confirmatory factor analysis (CFA; N = 200) [22]. The Kaiser–Meyer–Olkin (KMO) test was used to test the adequacy, and Bartlett’s Test of Sphericity was used to assess sphericity. KMO values between 0.7 and 0.8 were considered good and those between 0.8 and 0.9 were considered great [23]. The hidden factors were then extracted using principal axis factoring (PAF), varimax rotation, and the scree plot. The presence of one item in the factor was approximately 0.3 based on the following formula:

$${\text{CV}} = 5.152.\sqrt{(({\text{n}}-2))}.$$

CV is the number of extractable factors and n is the sample size of the study [24]. Factors were extracted by using first-order and second-order CFA (maximum likelihood estimation) and based on the commonly used goodness-of-fit statistics in structural equation modeling, including chi-square (w2), chi-square / degree-of-freedom ratio (normalized chi-square CMIN / DF), adjusted goodness-of-fit index (AGFI) > 0.8, Parsimonious Comparative Fit Index (PCFI) > 0.50, comparative fit index (CFI) > 0.90, incremental fit index (IFI) > 0.90, parsimonious normed fit index (PNFI) > 0.50, root mean square error of approximation (RMSEA) < 0.05 [25]. In the second-order factor analysis, it is assumed that the latent variables extracted in the first stage are themselves a reflection of another level of concept and can show a more general concept at the secondary and higher levels [26].

### Convergent and divergent validity

The convergent and divergent validity of the QoR-15 questionnaire was measured using Fornell and Larker (1981) approach and average variance extracted (AVE). To confirm convergent validity, AVE should be > 0.5, CR should be > 0.7, and CR > should be AVE [27], and to confirm divergent validity, the value square root of AVE for each factor must be greater than the value of the correlation coefficients of the factor with the other factors [28].

### Reliability

To evaluate the internal consistency of the QoR-15 questionnaire, Cronbach's alpha coefficient was estimated and values greater than 0.7 were considered appropriate [29]. Then, the construct reliability (CR) was calculated using confirmatory factor analysis. In fact, CR or factor stability is an alternative to Cronbach's alpha coefficient in structural equation modeling, and CR greater than 0.7 is considered appropriate. To determine the questionnaire's internal consistency, the test–retest reliability method was used recruiting 50 participants randomly selected from the total number of participants studied. This subset of patients(n = 50) was asked to repeat the QoR-15 a second time at around 30–60 min later and the correlation between measurements was assessed [29, 30]. There is no consensus about the length of time that should elapse between tests. Previous psychometric studies of the QoR-15 have been used the test–retest at 30 to 60 min later [12, 13]. Also, we believe that the 30–60-min time period used in this study was a sufficient duration that patients were unlikely to recall their previous answers., but not so long that actual changes in their postoperative health status had occurred.

## Results

### Demographic data

Among the participants (n = 450), 278 were males (61.7%), and 130 were over 55 years of age (28.9%). Other information is provided in Table 2.

**Table 2:** Demographic characteristics of the participants (n = 450)

Variable	N (%)
*Gender*	
Female	278 (61.7)
Male	172 (38.3)
*Age*	
< 25	66 (14.7)
25–35	68 (15.1)
35–45	83 (18.4)
45–55	103 (22.9)
> 55	130 (28.9)

### Content and face validity

The content validity of the Persian version of QoR-15 was judged to be good based on opinions from both patients and expert clinicians. According to Lawshe (1975), when the number of panelists is 15, the minimum acceptable CVR is equal to 0.49. All items received at least an acceptable ratio. Item content validity index (I-CVI) of each item was greater than 0.79. confirming its content validity. Further, this version was recognized as having good face validity in the sense of being clear, understandable, and easy to complete. All of the items in our QoR-15 questionnaire obtained an impact score greater than 1.5.

### Construct validity

**Fig. 1:**
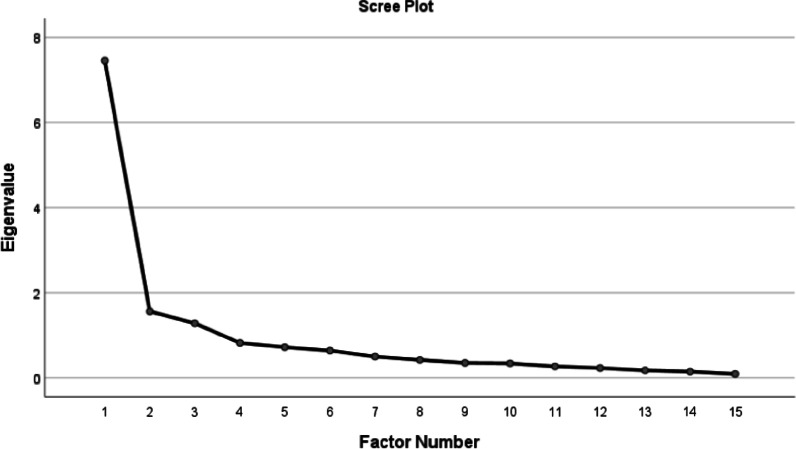
Scree plot for the EFA of the QoR-15 questionnaire

Sampling sufficiency index was calculated (KMO = 0.815 and Bartlett’s test = 1622.316, P < 0.001). In the scree plot (Fig. 1), factors with specific values greater than one were evaluated, and two factors, namely Part A [10 items] and Part B [5 items] were extracted in the EFA of the QoR questionnaire. These two hidden factors accounted for 4.631 and 3.565 of Eigenvalues specific value, respectively. In total, they explained 54.646% of the total variance of the QoR-15 questionnaire (Table 3). The factor loading of all items was greater than 0.4.

**Table 3:** Exploratory factors extracted from the QoR-15 questionnaire

Factor name	Items	Factor loading	*h^2^	Eigenvalues	% of variance
Part A	1. I was able to breathe easily	0.650	0.589	4.631	30.877
	2. I could enjoy the food	0.648	0.454		
	3. I had a feeling of vitality and liveliness	0.794	0.441		
	4. I had a good sleep	0.797	0.533		
	5. I was able to go to the bathroom without help and follow my routine personal hygiene	0.687	0.566		
	6. I was able to communicate with my family or friends	0.772	0.650		
	7. I was supported by the hospital's doctors and nurses	0.785	0.689		
	8. I was able to return to work or home routine activities	0.730	0.544		
	9. I felt comfortable and had control	0.747	0.668		
	10. In overall, I was satisfied and happy	0.676	0.702		
Part B	11. Moderate pain	0.554	0.425	3.565	23.769
	12. Severe pain	0.665	0.493		
	13. Nausea or vomiting	0.718	0.577		
	14. Feeling worried or anxiety	0.819	0.720		
	15. Feeling sad or depressed	0.693	0.547		

^*^ h^2^: Communalities

**Fig. 2:**
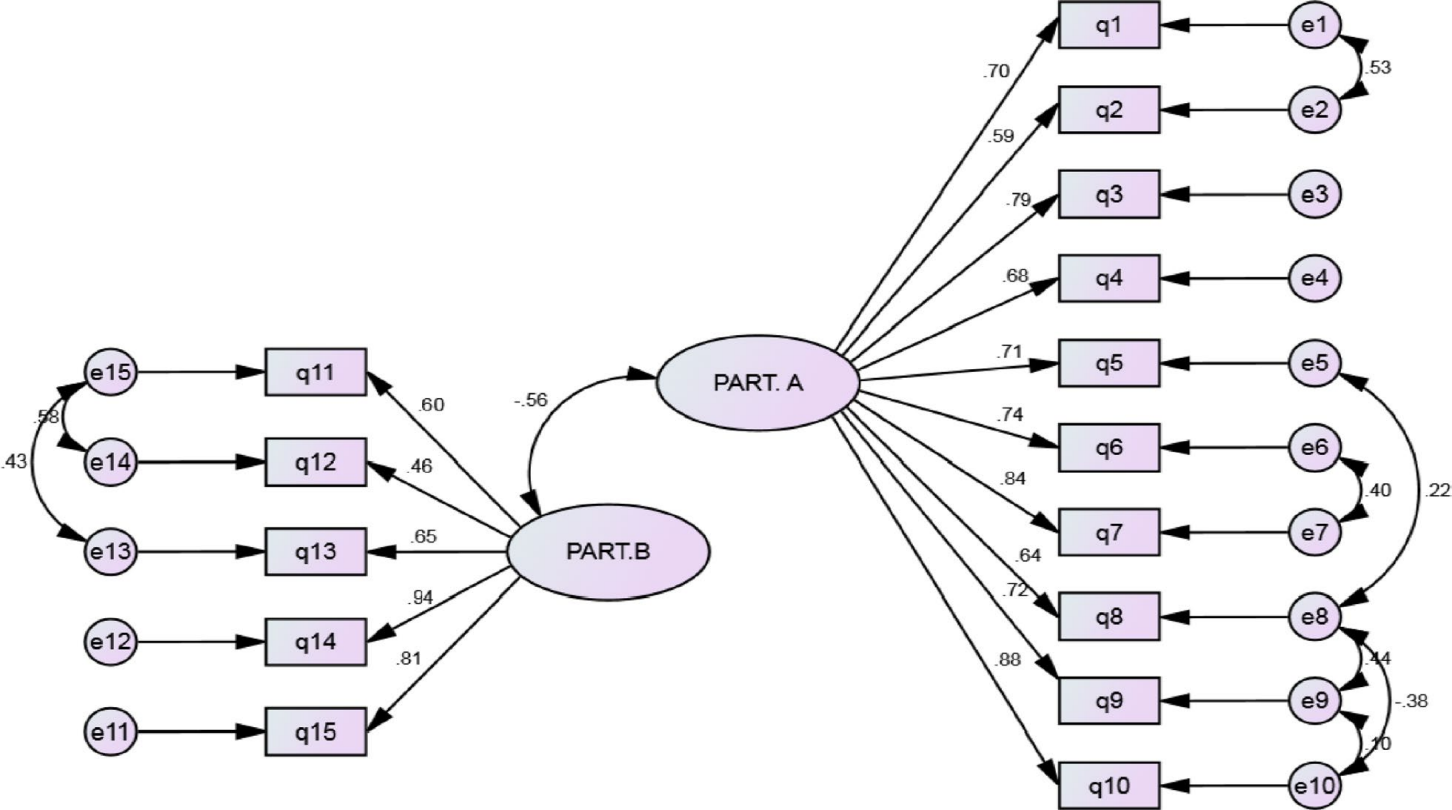
QoR-15 questionnaire: modified model of first-order CFA

The goodness-of-fit index, chi-square (p < 0.001), was obtained in first-order factor analysis:

χ2 (200) = 156.236. Then, other indices were examined to evaluate the model fit, all of which (RMSEA = 0.052, PCFI = 0.64, PNFI = 0.68, AGFI = 0.70, IFI = 0.92 and CFI = 0.96) confirmed the appropriate fitness of the final model (Table 4 and Fig. 2). After examining the first-order CFA model, the second-order factor analysis was performed using QOR-15 components separately and correlation between structures. The subscales were identified using structural equation modeling to evaluate whether the number of components is allocated in the overall concept of QOR-15.

**Table4:** Fit indices of first- and second-order confirmatory factor analysis of the QoR-15 questionnaire

CFA	χ2	df	P-value	CMIN/df	RMSEA	PCFI	PNFI	AGFI	IFI	CFI
First-order after structure modification	156.236	81	< 0.001	1.92	.052	.642	.681	.702	.924	.962
Second-order after structure modification	241.793	89	< 0.001	2.71	.062	.631	.573	.628	.903	.952

QoR-15: Quality of recovery; CFA: confirmatory factor analysis; CMIN/DF: Chi-square/degree-of-freedom ratio; RMSEA: Root Mean Square Error of Approximation; PCFI: Parsimonious Comparative Fit Index; PNFI: Parsimonious Normed Fit Index; AGFI: Adjusted Goodness-of-Fit Index; IFI: Incremental Fit Index; CFI: Comparative Fit Index

Fit indices: PNFI, PCFI, AGFI (> 0.5), CFI, IFI (> 0.9), RMSEA (> 0.08), and CMIN/DF (> 3 good, > 5 acceptable)

**Fig. 3:**
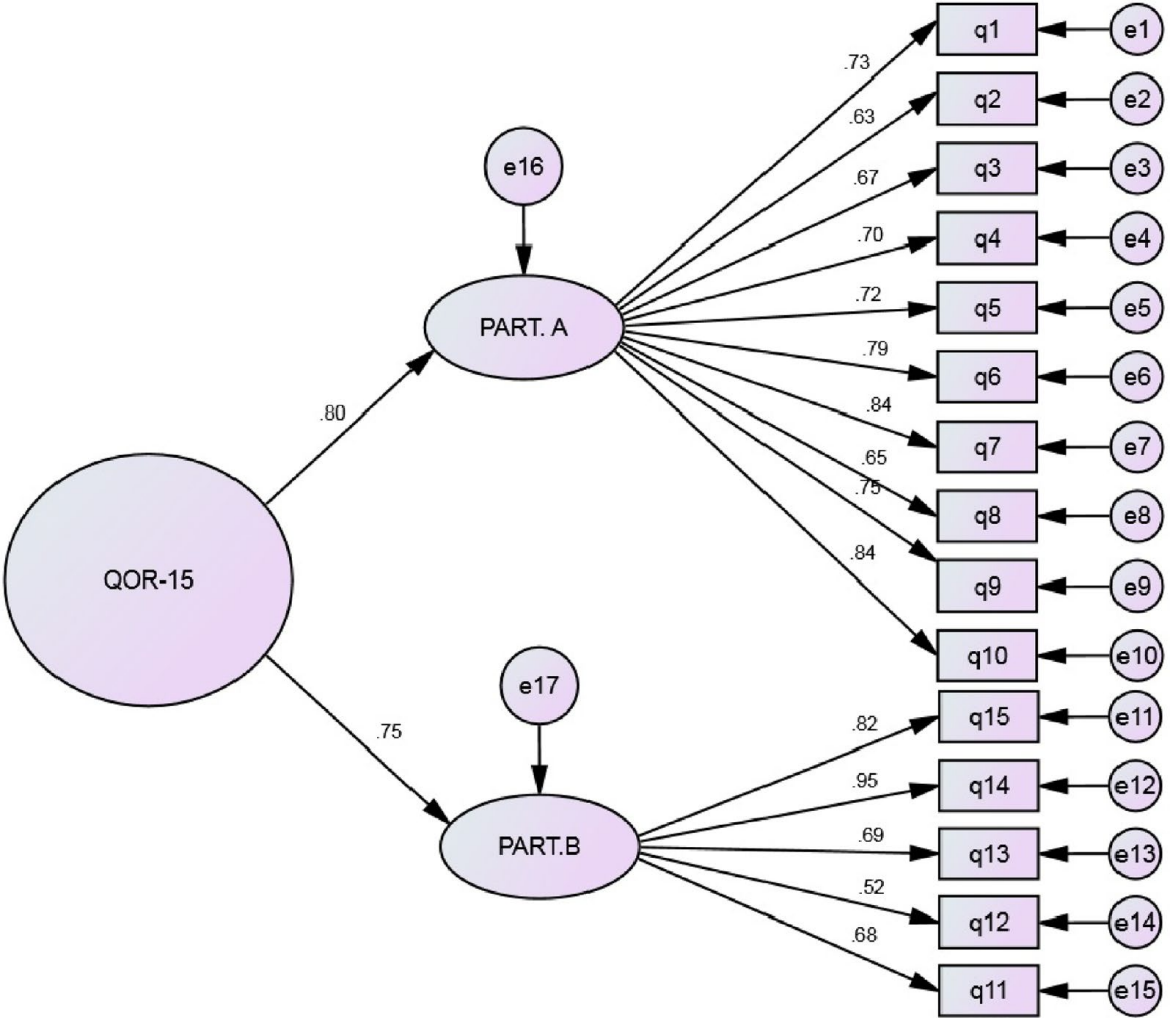
QoR-15 questionnaire: model of second-order CFA

The second-order confirmatory factor analysis fitness indices are shown in Table 4 in comparison with the first-order factor analysis model. Figure 3 shows the structural model and confirmatory factor analysis of the QoR-15 questionnaire in the factor loading mode with standardized coefficients. The amount of factor loading obtained for all QoR-15 items was greater than 0.5 and significantly lower than 0.001.

### Convergent and divergent validity

As can be seen in Table 5, in first-order confirmatory factor analysis, the values of CR and AVE for the two factors are greater than 0.7 and 0.5, respectively, and the value of AVE for each factor is greater than CR, suggesting good convergent validity. In addition, the value of the square root of AVE for both factors (on the diameter) is greater than the correlation coefficient between the two factors (not on diameter), revealing good divergence validity. The results showed that the QoR-15 questionnaire had appropriate convergent and divergent validity. In second-order factor analysis, AVE was greater than 0.5, confirming convergent validity.

**Table 5:** Internal consistency, composite reliability (CR), the square root of the average variance extracted (AVE) (in bold), and correlation between off-diagonal and QoR-15

Factors	ICC(95% CI)	P-value	α	CR	AVE	Factors	
						Part A	Part B
Part A	.843(.736–.913)	< .001	0.780	0.920	0.538	**0.733**	
Part B	.825(.775–.902)	< .001	0.851	0.829	0.506	− 0.601	**0.711**

^*^ Abbreviations; QoR: Quality of recovery; α: Cronbach's alpha coefficients; CR: Construct Reliability; AVE: Average Variance Extracted

### Reliability

According to Table 5, the internal stability and CR (> 0.7) of the four extracted factors from the QoR-15 questionnaire are confirmed. The test–retest reliability of the questionnaire was assessed using ICC. The means pre- and post-test scores of the questionnaire for the first factor were 61.64 ± 13.84 and 58.52 ± 3.84, respectively. ICC was equal to 0.843 (P < 0.001), and CI was equal to 0.736–0.913 at 95% confidence level. The means pre- and post-test scores of the questionnaire were 36.90 ± 5.99and 32.06 ± 3.12, respectively. ICC was equal to 0.825 (P < 0.001), and CI was 0.775–0.902 at the 95% confidence level (Table 5). To check further the reliability, CR and AVE indices of the convergent validity were examined for all variables and components. Both CR and AVE were greater than 0.7 and 0.5, respectively, for all variables and components. Accordingly, the convergent validity of the QoR-15 questionnaire was assured.

## Discussion

We conducted this study with the aim of translating the QoR-15 questionnaire into Persian and evaluating its psychometric properties in patients admitted to hospitals. This was the first study using the QoR-15 questionnaire in Iran. Based on our findings, the Persian version of the QoR-15 questionnaire can be a summary and clinically appropriate instrument to assess patients’ quality of recovery after surgery. Considering its good validity and reliability, this instrument is suggested in postoperative care in Iranian patients.

The high rate of responses showed that the QoR-15 questionnaire was acceptable and easy to complete for the patients. In addition, given that it is an outcome-based measure, it can enable nurses to manage postoperative care. In order to have a high return rate as well as decrease non-responders’ bias, patient-based outcome measures should be acceptable [11, 14]. Researchers in other countries also reported a high response rate while analyzing the psychometrically QoR-15 questionnaire, and concluded clinical usefulness of the QoR-15 questionnaire not only for patients but also for medical staff [11–13].

Unlike other QoR instruments such as QoR-40, QoR-15 is a short one and thus can be quickly studied and completed. Myles et al., generated and evaluated psychometrical characteristics of the 40-item instrument [10]. In Iran, Yaghoobi et al., in 2015, translated the 40-item QoR questionnaire (QoR-40) to Persian and measured its psychometric properties in 200 patients after general anesthesia. They reported that the Persian version of the QoR-40 questionnaire could be a valid and reliable instrument to assess the recovery quality after surgery in Iranian patients [7]. However, it is a lengthy questionnaire and often takes about ten minutes for patients to fill this instrument. Unlike the QoR-40 questionnaire, the QoR-15 questionnaire can be completed in less time [5]. Similarly, it was yielded that the Swedish version of the QoR-15 questionnaire was completed by patients in less time [13]. In line with previous studies, patients completed the QoR-15 questionnaire in a good average time in this study. This time was longer in our study in comparison with that of the original one. The reason is that some of the patients who were elderly and disabled did not complete the QoR-15 questionnaire themselves and the researcher completed the questionnaire for them.

The QoR-15 questionnaire is easy to complete and suitable to use. It is validated to detect clinically meaningful health status changes based on the patient’s understanding [4, 13]. Noll et al., (2017) showed that the QoR-15 questionnaire was appropriate for evaluating the effectiveness of acupressure therapy on QoR and satisfaction among patients. They also suggested the QoR-15 questionnaire as a suitable instrument to assess self-rated overall health status after surgery [4].

Internal consistency was measured using Cronbach’s alpha coefficient, test re-test, and CR, and acceptable reliability (> 0.78) was observed. similarly, Kleif et al., (2015) reported Cronbach’s alpha 0.88 for the QoR-15 questionnaire [11]. In British, Chazapis et al., in 2016, measured the QoR-15 questionnaire internal consistency and found high and satisfied Cronbach’s alpha (0.70–0.90) [12]. Moreover, internal consistency was calculated in this study based on inter-item correlation. Accordingly, the average inter-item correlation for parts A and B of the QoR-15 questionnaire was 0.73 and 0.71, respectively, revealing that both parts were well correlated with the overall QoR-15 questionnaire.

The QoR-15 questionnaire demonstrated strong construct validity with CFA in the present study. Factor load of some items was more than 0.05. The highest factor load in factor 1 was related to the “*I had a good sleep*.” item. The least was related to the “*I could enjoy the food*” item. Accordingly, it seems that patients paid more attention to sleep and rest within the first 24 h after surgery. The second factor, part B, includes 5 items. The highest factor load was related to the “*Feeling worried or anxiety*” item. The least was allocated to the “moderate pain” item. In addition to the experience of severe pain within the first 24 h after surgery, patients had concerns about their recovery, which seems normal.

Previous studies have reported that most patients returned to good recovery 48 h after surgery, but in some cases did not have adequate recovery quality for up to seven days after surgery. Therefore, it is not possible to consider a limited time to use this scale. But symptoms like severe pain will decrease over time. Previous studies have reported severe pain relief after 48 h and then 7 days. Anxiety and worry have also decreased over time. In our study, most patients experienced severe pain 24 h after surgery.

Question one, the ability to breathe easily, and question six, the ability to communicate with all of family or friends, had the highest response rates. On the other hand, items of ‘the ability to maintain personal toilet and hygiene unaided’ and ‘the ability to return to work or usual home activities’ had the lowest response rates. Given that these two items address important parts of a patient’s recovery and wellbeing, the reason for their low response rates must be identified. It may be due to the unexpectedness of reaching these two items 24 h later surgery from of Iranian patients’ perspective.

Some limitations should be considered when interpreting the results of this study. First, our study was conducted in patients undergoing general and orthopedics surgery with limited demographic information in the hospitals of Tehran University of Medical Sciences and its generalization should be done with caution. Second, some patients who did not complete the questionnaire themselves may cause bias in results. To overcome some of these limitations, we recommend future studies be conducted on patients with diverse demographic characteristics in various health centers and the questionnaire be filled out by the patients themselves.

## Conclusion

The QoR-15 questionnaire was a reliable and valid instrument for measuring the quality of recovery 24 h after surgery. In this study, the patients completed the QoR-15 questionnaire 24 h after surgery, but not immediately after surgery. It was found that the QoR-15 questionnaire can be a useful and feasible tool to assess patients’ outcomes after surgery. In accordance with the findings of the current investigation, the QoR-15 questionnaire can be implemented in clinical settings to measure the effect of interventions on Iranian healthcare delivery. Given that it is simple and short-scale; it can also enable nurses to provide postoperative care tailored to patients' needs.

## Data Availability

The datasets used during the current study are available from the corresponding author on reasonable request.

## Abbreviations

QoR-15: Quality of recovery-15
EFA: Exploratory factor analysis
CFA: Confirmatory factor analysis
CVR: Content validity ratio
CVI: Content validity index
I-CVI: Item content validity index
S-CVI: Scale content validity index
KMO: Kaiser–Meyer–Olkin
PAF: Principal axis factoring
CV: Construct validity
AVE: Average variance extracted
CR: Construct reliability
α: Cronbach's alpha coefficients
